# Effects of Grass Carp Antifreeze Peptide on Freeze-Thaw Characteristics and Structure of Wet Gluten Protein

**DOI:** 10.3390/foods14244336

**Published:** 2025-12-16

**Authors:** Meizhu Dang, Bing Huang, Yangyang Jia, Yuanyuan Shao, Xingxing Mei, Chunmei Li

**Affiliations:** 1 Livestock Products Quality Surveillance Institute, Henan University of Animal Husbandry and Economy, Zhengzhou 450002, China; 80423@hnuahe.edu.cn; 2 College of Food Science and Technology, Huazhong Agricultural University, Wuhan 430072, China; 3 School of Energy and Intelligence Engineering, Henan University of Animal Husbandry and Economy, Zhengzhou 450002, China; huangbing_10@126.com; 4 School of Food Science, Henan Institute of Science and Technology, Xinxiang 453003, China; 5 College of Materials and Chemical Engineering, Zhengzhou University of Technology, Zhengzhou 450002, China; 6 School of Economics and Trade, Henan University of Animal Husbandry and Economy, Zhengzhou 450002, China

**Keywords:** grass carp antifreeze peptide, water mobility, ice recrystallization inhibition, protein structural stability, rheological behavior, frozen food preservation

## Abstract

This study uniquely explores the impact of a novel natural antifreeze peptide derived from grass carp (GCAFP) on the freeze–thaw characteristics and structural stability of wet gluten protein, providing new insights into the development of natural cryoprotectants for frozen foods. The effects of GCAFP on the physicochemical and structural properties of gluten protein were investigated using differential scanning calorimetry (DSC), nuclear magnetic resonance imaging (NMR), rheology, and scanning electron microscopy (SEM). The results showed that the addition of 0.5% GCAFP significantly reduced the freezing temperature (T_f_, from −8.50 ± 1.31 °C to −10.75 ± 2.49 °C) and expanded the melting temperature range (T_m,δ_, from 3.60 ± 1.40 °C to 5.65 ± 0.12 °C), indicating improved freezing stability. After five weeks of frozen storage, the ice crystal melting enthalpy (ΔH_m_) of gluten protein in the GCAFP group increased by only 20.17 J/g, compared with 27.23 J/g in the control, representing a 6.35% reduction (*p* < 0.05). Similarly, after five freeze–thaw cycles, the freezable water fraction (F_w_) and ΔH_m_ were reduced by 5.19% and 1.55%, respectively, demonstrating that GCAFP inhibited water migration and ice recrystallization. Low-field NMR revealed that GCAFP maintained a higher proportion of bound water (T21) and decreased free water (T23), confirming its role in restricting water mobility. Rheological analysis showed that GCAFP preserved the viscoelasticity of gluten protein, maintaining higher storage (G′) and loss (G″) moduli than the control after five freeze–thaw cycles, thus mitigating the decline in network elasticity. Structural characterization indicated that GCAFP stabilized the α-helix and β-sheet contents, reduced glutenin macropolymer depolymerization from 24.85% to 18.95%, and strengthened hydrogen bonding within the protein matrix. Overall, GCAFP effectively protected wet gluten protein against ice crystal damage by maintaining water distribution, viscoelasticity, and secondary structure integrity, highlighting its potential as a natural antifreeze ingredient for frozen food applications.

## 1. Introduction

Recently, antifreeze proteins and antifreeze peptides have attracted growing attention as functional additives for improving the quality of frozen dough and gluten-based products [1,2]. Antifreeze proteins exhibit thermal hysteresis activity and can modify ice crystal morphology while inhibiting ice crystal recrystallization, thereby preserving the structural integrity of frozen food systems [3]. Beyond their antifreeze function, antifreeze proteins and peptides also contribute to nutritional enhancement and textural improvement in frozen dough [4]. During frozen storage, temperature fluctuations induce the formation and recrystallization of ice crystals, causing mechanical damage to the gluten network and reducing the dough’s gas retention capacity. Ice crystals can also puncture yeast cells, leading to reduced yeast viability and deteriorated product quality. AFPs mitigate these effects by controlling ice crystal growth and recrystallization [5,6], maintaining the structural and functional properties of wet gluten protein during frozen storage. For example, Kontogiorgos et al. investigated the impact of antifreeze proteins, also known as Ice-structuring proteins (ISPs), on the flour–water system and the wet gluten protein system during freezing and found that ISPs shielded the system from damage to the gluten network caused by ice crystal formation and recrystallization [7,8]. Li et al. found that winter wheat ISPs effectively reduced ice crystal formation and recrystallization by lowering freezable water content (F_w_) in wet gluten protein, thereby preserving its network structure during frozen storage [9]. This ultimately compromises frozen Mantou quality by lowering the specific volume and increasing hardness. Sun et al. revealed that salmon antifreeze peptide (SaAFP) bound to ice crystal surfaces via hydrophilic amino acid side chains, thus inhibiting ice growth and recrystallization, which reduced ice-induced gluten damage, improved yeast survival, and significantly enhanced gas production in frozen fermented dough. Mantou treated with SaAFP exhibited higher specific volume, lower hardness, and improved overall quality [10]. Yanjie Zhang et al. examined the thermal and rheological properties and microstructure of hydrated gluten as influenced by oat antifreeze protein (AsAFP). The results showed that the addition of AsAFP increased the glass transition temperature and decreased the melting enthalpy and freezable water content of fresh hydrated gluten. The supplementation of AsAFP also influenced the melting performance of hydrated gluten after freeze–thaw treatment [11].

Our previous study identified novel, natural AFPs from *Ctenopharyngodon idella* scales. Molecular dynamic simulations revealed that GCAFP disrupted ice-water interactions, thereby inhibiting ice crystallization, suggesting its potential as a safe and effective antifreeze agent for the food industry [12]. However, its actual effect on the characteristics or structure of gluten protein is unclear. Therefore, this study aimed to further explore its effects on gluten protein, a key component of pre-fermented frozen dough, and its potential application in frozen dough products. The effects of GCAFP on thermodynamic properties, water status, rheology, and microstructure of wet gluten protein were investigated. We believe that our data will establish a scientific basis for further applying GCAFP, in frozen dough products.

## 2. Materials and Methods

### 2.1. Materials and Chemicals Instruments

Grass carp scales were purchased from a local market in Wuhan, China. Gluten (protein content 77.42%) was obtained from Henan Luohe Xuejian Industrial Co., Ltd. (Luohe, China). ANS was sourced from Sangon Biotech (Shanghai) Co., Ltd. (Shanghai, China). All other reagents were of analytical grade unless otherwise specified and were purchased from Shanghai Chemical Reagent Company of China National Pharmaceutical Group (Shanghai, China).

### 2.2. Preparation of Wet Gluten Protein

The antifreeze peptide derived from grass carp (GCAFP) was prepared following a previously published method. Briefly, grass carp (Ctenopharyngodon idella) scales were cleaned, crushed, and decalcified in 1 mol/L citric acid solution for 24 h. The pretreated scales were hydrolyzed using selected proteases (neutral protease, alcalase, papain, trypsin, and pepsin) under their respective optimal conditions. The peptide hydrolysates were evaluated based on their total hydrophobic amino acid content, and the fraction exhibiting the highest antifreeze activity was collected, purified, and lyophilized to obtain GCAFP powder.

Wet gluten protein was prepared according to a modified procedure described previously [13]. For the control group, wheat gluten and distilled water were mixed at a 1:1 (w/w) ratio to hydrate and form a cohesive gluten network. For the GCAFP-treated group, wheat gluten protein and 0.5% GCAFP (w/w, based on gluten) were vortexed and mixed with water at the same ratio to hydrate and form gluten. The mixture was equilibrated at 4 °C for 1 h to obtain wet gluten protein (50% w/w). Some samples were sealed and packaged for direct freezing, while others were accurately weighed and frozen in DSC aluminum pans for thermal analysis. All samples requiring frozen storage were placed at −35 °C for 2 h and then stored at −20 °C. After frozen storage and freeze–thaw cycles, selected samples were analyzed, while others were freeze-dried, ground, and sieved using a 0.25 mm mesh for further analysis.

### 2.3. Apparent Specific Heat of Wet Gluten Protein

The apparent specific heat of wet gluten protein was determined using a previously reported method [14]. This parameter represents the specific heat during the phase change of food and is closely related to its structure and energy. It serves as a fundamental factor in designing frozen storage facilities and analyzing thermal stress during freezing [15]. Wet gluten protein was used for measurement, with KCl as the standard substance. The standard specific heat value of KCl was obtained from the literature [16].

A 5 mg sample of wet gluten protein was placed in a DSC crucible, sealed with a lid, and positioned in the sample pool of the instrument furnace. The initial temperature was set to 20 °C, then reduced to −60 °C at a rate of 5 °C/min, followed by an increase to 10 °C at a rate of 1 °C/min. The heat flow of an empty crucible was measured using the same procedure as DSC_b_.

The apparent specific heat (C_app_) was calculated using the following equation:

(1) $$C_{\mathrm{app}}=\frac{m_{std}}{m_{s}}\cdot \frac{DSC_{S}-DSC_{b}}{DSC_{Std}-DSC_{b}}\cdot C_{p,std}$$

where *C*_app_ represents the apparent specific heat of wet gluten protein (J/g·°C); m_std_ is the weight of the standard substance KCl (mg); ms is the sample weight (mg); DSC_S_ is the heat flow of the sample (M_w_); DSC_b_ is the heat flow of the control group (M_w_); DSC_std_ is the heat flow of the standard substance KCl (M_w_); C_p,std_ is the apparent specific heat of the standard substance KCl (J/g·°C).

### 2.4. Freeze–Thaw Characteristics of Wet Gluten Protein

The freeze–thaw characteristics of wet gluten protein include freezing temperature (T_f_), melting temperature (T_m_), melting enthalpy (∆H_m_), and freezable water content (F_w_) [14]. Each sample (about 10 mg) was cooled to −90 °C at a rate of 10 °C/min, using an empty plate as a reference, and equilibrated for 5 min. The sample was then heated to 20 °C at 10 °C/min before being cooled to −90 °C at the same rate. The sample was further cooled to −20 °C at 10 °C/min, annealed for 30 min, then cooled to −90 °C at 5 °C/min, and finally heated to 20 °C at 2 °C/min. T_f_ was defined as the peak temperature of the freezing curve, while T_m,o_ was the onset temperature of the heat absorption curve. The peak temperature (T_m,p_) and the end temperature (T_m,e_) of the melting curve were also determined, and the melting temperature range (T_m,δ_) was calculated. F_w_ was calculated as the ratio of frozen water to total water:

(2) $$F_{W}\left(\%\right)=\frac{\Delta H_{W}}{\Delta_{fus}H_{m}m\times W_{A}}\times 100$$

where ${∆H}_{W}$ refers to the melting enthalpy of the sample (J); ${{∆}_{fus}H}_{m}m=3$33.3 J/g; m refers to the sample mass (g); $W_{A}$ refers to the water content of the sample (g/g).

### 2.5. Melting Characteristics and Fw of Wet Gluten Protein

The melting characteristics and F_w_ of wet gluten protein were determined using DSC (Netzsch, Selb, Germany, DSC204-F1). Sample processing followed a previously reported method [17]. A 10 mg sample from the center of frozen dough stored for various durations was sealed in a DSC aluminum crucible and weighed. To prevent aging and condensation from ice crystal melting, the DSC aluminum crucible was transferred from low-temperature storage to the sample pool at an initial temperature of −30 °C. The sample was sealed in a DSC aluminum crucible and maintained at −30 °C until testing [8]. During the test, the sample was equilibrated at −30 °C for 5 min, then transferred to a precooled DSC chamber at −30 °C. The temperature was increased to 15 °C at a rate of 1 °C/min, and the melting curve was recorded. Thermal analysis software used ∆Hm to calculate F_w_.

### 2.6. Water Fluidity and Distribution of Wet Gluten Protein

The water fluidity and distribution of pre-fermented frozen dough were measured using a previously reported method [18]. The transverse relaxation time (T_2_) was determined using low-frequency NMR (NMI20-025V-I, Suzhou, China). The resonant frequency was 21 MHz, magnetic field strength was 0.5 T, coil diameter was 60 mm, and magnet temperature was 32 °C. The sequence parameters were as follows: 90° pulse time (P_90_), 14 µs; 180° pulse time (P_180_); 35 µs; sampling points (TD), 67,042; main frequency (SW), 200 kHz; repetition time (TR), 500 ms; analog gain (RG_1_), 20; analog gain (RG_2_), 3; accumulation times (NS), 8; echo number (NECH), 1000; and echo time (TE), 0.335 ms.

A 1.00 ± 0.01 g sample was accurately weighed, wrapped in plastic film, and placed in an NMR tube. The tube was positioned at the center of the RF coil within the permanent magnetic field for a Carr–Purcell–Meiboom–Gill (CPMG) pulse sequence scanning experiment using low-field nuclear magnetic analysis.

Test conditions for water fluidity were as follows: sampling frequency, 100 kMHz; repeated sampling times (NS), 8; half echo time (τ), 0.25 ms; repetition time (TR), 1000 ms; sampling points, 199,990; relaxation decay time (T_0_), 1500 ms. The relaxation time peak area was expressed as the response value peak area per gram of sample. The CPMG relaxation decay curve was analyzed using T_2_ FitFrm software to obtain spectra and T_2_ values for each sample.

Low-field MRI conditions: imaging thickness, 5 mm; FOVRead, 100 mm; FOVPhase, 80 mm; TR, 500 ms; slices, 2; averages, 4; slice width, 3.0 mm; and TE, 20 ms. Pseudo-color processing was applied after proton density image acquisition.

### 2.7. Dynamic Rheological Characteristics of Wet Gluten Protein

A rotational rheometer (DHR-2, TA Instruments, New Castle, DE, USA) was used to perform frequency sweeps in oscillatory mode to investigate the impact of frozen storage on the rheological properties of wet gluten protein. After frozen storage, the wet gluten protein was thawed at room temperature for 1 h before measurement. The probe had a 20 mm diameter, the gap distance was 2 mm, and the sample was placed between the plates at 25 °C, allowed to relax for 5 min, and excess material was removed. The deformation (strain) was set at 0.5%, which was within the linear viscoelastic region (LVR) determined by preliminary strain sweep tests. The frequency ranged from 0.01 to 40 Hz, and the temperature was maintained at 25 °C.

### 2.8. Structural Characterization of Wet Gluten Protein

#### 2.8.1. Content of Free S-H in Wet Gluten Protein

Contents of free S-H and total S-H were measured using previously reported methods [19,20].

Free S-H content estimation: A 30 mg protein sample was weighed, mixed with 2 mL of extraction buffer (containing 2.5% SDS (Sodium dodecyl sulfonate), 92 mmol/L glycine, 4.1 mmol/L EDTA (Ethylene Diamine Tetraacetic Acid), and 86 mmol/L Tris-HCl (Tris (hydroxymethyl) aminomethane hydrochloride), pH 8.0), and vortexed. The mixture was shaken at room temperature (200 r/min) for 60 min, centrifuged (12,000× *g*, 20 °C, 10 min), and 150 μL of the supernatant was collected. To this, 2.5 μL of DTNB (5,5′-Dithiobis(2-nitrobenzoic acid) solution (4 mg/mL) (sample) or 2.5 μL of extraction buffer (control) was added. The reaction proceeded in the dark at room temperature for 30 min. Absorbance at 412 nm was measured using the equation A412 nm = Asample − Acontrol. Reduced glutathione was used as the standard for quantifying free thiol (S-H) content.

Total S-H content estimation: A 15 mg protein sample was accurately weighed and suspended in 2 mL of 80 mmol/L Tris-HCl buffer (pH 8.5) containing 40 mmol/L DTT (Dithiothreitol). The mixture was oscillated (200 r/min) at 60 °C for 2 h following vortexing. Then, 1.2 mL of pre-cooled (−20 °C) 100 mmol/L glacial acetic acid-acetone reagent was added. After mixing, the sample was centrifuged (5000× *g*, 5 min), and the supernatant was discarded. The precipitate was resuspended in 200 μL of 100 mmol/L acetic acid solution, vortexed to dissolve the protein, and mixed with 2 mL of pre-frozen (−20 °C) acetone. The sample was centrifuged (5000× *g*, 5 min), and the washing step was repeated. The precipitate was then resuspended in 2 mL of extraction buffer (containing 2.5% SDS, 92 mmol/L glycine, 4.1 mmol/L EDTA, and 86 mmol/L Tris-HCl, pH 8.0). After vortexing, the mixture was oscillated (200 r/min) for 60 min at room temperature and centrifuged (12,000× *g*, 20 °C, 10 min). A 10 μL aliquot of the supernatant was mixed with 140 μL of extraction solution. Then, 2.5 μL of DTNB solution (4 mg/mL) (sample) or 2.5 μL of extraction buffer (control) was added. The reaction was conducted at room temperature in the dark for 30 min. Absorbance at 412 nm was measured. The total S-H content was quantified using reduced glutathione as the standard.

#### 2.8.2. Secondary Structure of Wet Gluten Protein

The secondary structure of wet gluten protein was analyzed using a Thermo Scientific^TM^ Nicolet^TM^ iS50 FTIR spectrometer (Thermo Fisher Scientific, Waltham, MA, USA). A measured amount of protein was placed in an agate crucible and mixed with diluent KBr. The mixture was ground evenly and pressed into a pellet for measurement. The test parameters included 32 scans, a spectral range of 400–4000 cm^−1^, and a resolution of 4 cm^−1^. The amide I region (1600–1700 cm^−1^) was examined using OMNIC 8.0 and PeakFit 4.12. Baseline calibration, smoothing, deconvolution (Gaussian method), and the second-order derivative method were applied to fit the curves. The content of each secondary structure was determined based on its respective peak area.

#### 2.8.3. SDS-PAGE of Wet Gluten Protein

Protein Extraction: Wet gluten protein (80 mg) was accurately weighed into an Eppendorf tube and mixed with 500 μL of lysis buffer containing 1 mM PMSF. Samples were homogenized six times using a ferromagnetic bead grinder and incubated at 4 °C for 60 min with gentle inversion. Ultrasonic disruption was then performed on ice (5% amplitude; 2 s on, 2 s off, for a total of 1 min). Samples were centrifuged at 17,000× *g* for 15 min at 4 °C, and 380 μL of the supernatant was collected. Proteins were precipitated by adding 1520 μL of pre-cooled methanol-acetonitrile solution (1:1, v/v) and incubated at −20 °C overnight. After centrifugation (17,000× *g*, 15 min, 4 °C), the supernatant was discarded, and the pellet air-dried. The pellet was resuspended in 100 μL of lysis buffer and sonicated under the same conditions. After a second centrifugation (17,000× *g*, 15 min, 4 °C), the supernatant was collected. A 30 μL aliquot of the supernatant was mixed with 6 μL of 6× reducing SDS-PAGE loading buffer, vortexed, briefly centrifuged, and heated at 99 °C for 8 min. The sample was then briefly centrifuged, and the supernatant loaded onto the gel.

SDS-PAGE Electrophoresis: Protein separation was performed using a 12% resolving gel and a 5% stacking gel. Tris-glycine-SDS buffer (pH 8.3) was used as the running buffer. Samples (30 μL) were loaded into wells of the stacking gel. Electrophoresis was conducted at 100 V for the stacking gel and 120 V for the resolving gel until the bromophenol blue dye front reached the bottom (DYY 6C, Liuyi, China) [21].

Staining and Imaging: Gels were stained overnight with Coomassie Brilliant Blue solution (3 g/L) and subsequently destained in a solution containing acetic acid, methanol, and distilled water (10:45:45, v/v/v) until protein bands were clearly visible. Protein bands were imaged using a Gel Doc^TM^ EZ Imaging System (Bio-Rad Laboratories, Inc., Hercules, CA, USA) for documentation and analysis.

#### 2.8.4. Distribution of Relative Molecular Weight in Wet Gluten Protein

The molecular weight (M_w_) of gluten protein was measured using an Agilent 1200 HPLC system (Agilent Technologies, Santa Clara, CA, USA) equipped with a Shodex Protein KW-804 column (Showa, Kyoto, Japan). At room temperature, 10 mg of lyophilized sample was extracted with 5 mL of phosphate-buffered saline (PBS; 0.05 M, pH 6.8, 2.0% SDS) for 5 h, followed by centrifugation (10,000× *g*, 5 min). The supernatant was filtered through a 0.45 μm membrane, and 20 µL of the filtrate was injected into the column under the following conditions: column temperature, 30 °C; flow rate, 0.7 mL/min; detection wavelength, 214 nm. The peak area of extracted proteins was determined as the total protein peak area using an SDS buffer containing 1.0% dithiothreitol (DTT). The percentages of total protein peak areas were used to quantify SDS-soluble polymer (SDS-P), SDS-soluble monomeric protein (SDS-M), and SDS-insoluble protein (SDS-I).

#### 2.8.5. Microstructure of Wet Gluten Protein

Samples were cut into uniformly sized pieces from the center, gold-sputtered for 60 s, and examined under a field-of-view magnification of 300× and 1200× [18].

### 2.9. Statistical Analysis

Results are expressed as means ± standard deviation. Statistical analysis was performed using the Kruskal–Wallis test (SPSS 22.0), with significance defined at *p* < 0.05. All experiments were conducted in triplicate. All measurements in this study were performed in triplicate unless otherwise specified.

## 3. Results and Discussion

### 3.1. Effects of GCAFP on Thermodynamic Properties of Wet Gluten Protein

#### 3.1.1. Effects of GCAFP on the Apparent Specific Heat of Wet Gluten Protein

**Figure 1:**
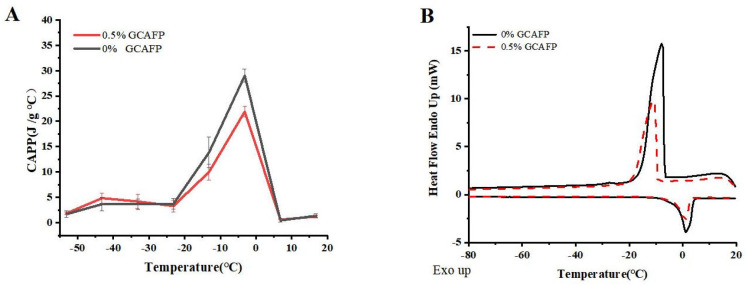
Effect of GCAFP on apparent specific heat and freeze–thaw parameters of wet gluten protein. (**A**) Effect of GCAFP on apparent specific heat of wet gluten protein; (**B**) Effect of GCAFP on freeze–thaw parameters of wet gluten protein. Note: GCAFP: Grass carp antifreeze peptide.

Figure 1A illustrates the effects of GCAFP on the apparent specific heat of wet gluten protein. Similar to dough, wet gluten protein exhibits latent heat variations during phase transitions. Within the temperature range of −60 to 20 °C, distinct phase transitions occurred, leading to notable fluctuations in apparent specific heat. At low temperatures (−60 °C to the freezing point), the apparent specific heat gradually increased with rising temperature, particularly between −15 °C and 1 °C. Before the onset of phase transition (at −53.15 °C), the apparent specific heat of the control and GCAFP samples was 1.67 ± 0.61 and 1.86 ± 0.23 J/(g·°C), respectively. Across the range of −53.15 to 33.15 °C, the apparent specific heat of the GCAFP-treated samples consistently remained higher than that of the control. During the phase transition, as the temperature increased to −3.15 °C, the apparent specific heat of GCAFP-treated sample reached 29.02 ± 1.31 J/(g·°C), which was significantly higher than that of the control (21.91 ± 1.02 J/(g·°C), *p* < 0.05). After the transition, ice crystals had completely transformed into liquid water, eliminating the influence of latent heat; thus, further temperature increases caused only minimal changes in specific heat. Consequently, further temperature increases resulted in minimal changes in apparent specific heat. Compared with dough, the apparent specific heat of wet gluten exhibited greater variability. Wet gluten protein, an essential dough component, has lower hydrophilic content and higher water fluidity, allowing it to release substantial heat rapidly during freezing. This suggests that the influence of GCAFP on the apparent specific heat of gluten protein plays a key role in its overall effect on dough freezing behavior. The addition of GCAFP enhanced water fluidity within the gluten matrix, slowing water migration. As a result, freezing became more difficult in the GCAFP group, and the progression of apparent specific heat changes was more gradual from a thermodynamic perspective.

#### 3.1.2. Effects of GCAFP on the Freeze–Thaw Characteristics of Wet Gluten Protein

Figure 1B depicts the freeze–thaw behavior of wet gluten protein, and the corresponding thermodynamic parameters are summarized in Table 1. The freeze–thaw curves indicate that, after GCAFP addition, the peak freezing temperature of wet gluten protein decreased compared with the control, and the melting temperature range narrowed significantly. These results clearly indicate that GCAFP influenced the freezing behavior of gluten protein. Specifically, upon GCAFP addition, T_f_ of wet gluten protein decreased from −8.50 ± 1.31 °C to −10.75 ± 2.49 °C, T_m,o_ dropped from −1.70 ± 0.41 °C to −2.05 ± 0.37 °C, and T_m,p_ fell to −1.15 ± 0.04 °C, indicating that GCAFP addition reduced key temperature parameters in the wet gluten protein system significantly (*p* < 0.05). This finding aligned with previous studies [14], further confirming the effectiveness of GCAFP in regulating the freezing system temperature. T_m,δ_ represents the difference between T_m,e_ and T_m,o_ of the melting curve and reflects the gluten protein melting temperature range. As shown in Table 1, the T_m,δ_ of the GCAFP-treated gluten protein sample increased significantly from 3.60 ± 1.4 °C to 5.65 ± 0.12 °C (p). The increase in T_m,δ_ suggested that GCAFP widened the melting temperature range of gluten protein, suggesting that the transition from frozen to molten state was more gradual. Consequently, during frozen storage, GCAFP effectively mitigated gluten protein quality deterioration caused by temperature fluctuations, thus enhancing its freezing stability.

According to Table 1, the ΔH_m_ and F_w_ values of the control sample showed an increasing trend, while those of the GCAFP-treated gluten protein decreased. This trend resembled the effect of GCAFP in the dough, suggesting that during the freeze–thaw process, the hydrophilic component of GCAFP bound with free water in the gluten protein. This interaction enhanced water retention, reduced water mobility, and consequently decreased F_w_ [22]. Since the amount of freezable water is directly correlated with ice crystal formation, and ice crystal growth and migration can severely disrupt the gluten network [23], the presence of GCAFP effectively protected the gluten protein network during freezing, improving gluten protein quality throughout the freeze–thaw cycle.

#### 3.1.3. Effects of GCAFP on the Melting Characteristics and Fw of Wet Gluten Protein

**Figure 2:**
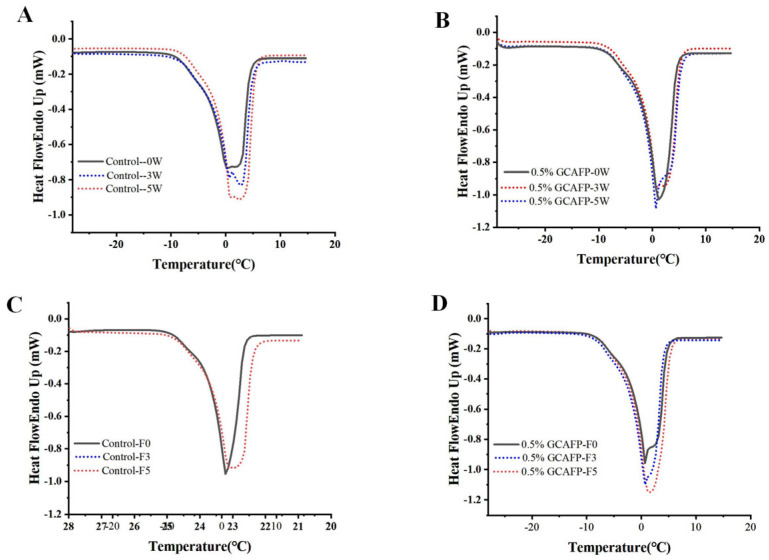
Effect of GCAFP on melting parameters of wet gluten protein. (**A**) 0% GCAFP 0, 3, 5 after freeze treatment; (**B**) 0.5% GCAFP 0, 3, 5 after freeze treatment; (**C**) 0% GCAFP 0, 3, 5 freeze–thaw cycles; (**D**) 0.5% GCAFP 0, 3, 5 freeze–thaw cycles. Note: GCAFP: Grass carp antifreeze peptide.

Figure 2A,B presents the melting curves of wet gluten protein following frozen storage. Compared with those of the freeze–thawed samples, the melting curves exhibited noticeable shifts. After both 3 and 5 weeks of frozen storage, the degree of melting curve shift in the control group was greater than that observed in the GCAFP group.

The melting parameters of wet gluten protein after different storage durations were analyzed using Pyris 1 software to determine ice crystal melting enthalpy (Table 2). The results revealed that after 5 weeks of frozen storage, the ice crystal melting enthalpy of gluten in the control sample increased from 96.91 ± 2.47 to 124.14 ± 4.76 J/g (an increase of 27.23 J/g), whereas the introduction of 0.5% GCAFP resulted in an increase from 96.08 ± 1.30 to 116.25 ± 2.40 J/g (a rise of 20.17 J/g). This indicates that gluten ice crystal melting enthalpy gradually increased with extended storage, with the most rapid change occurring within the first 5 weeks, consistent with previous studies [24,25]. Further analysis showed that the introduction of 0.5% GCAFP significantly (*p* < 0.05) reduced ΔH_m_ of gluten protein ice crystals by 6.35% compared with the control after 5 weeks, suggesting that GCAFP effectively inhibited ice crystal growth. Similarly, Kontogiorgos and Goff reported that wheat ISPs significantly reduced ice crystal melting enthalpy of frozen wet gluten protein [7,8].

T_m,o_ of the control sample increased from −3.35 ± 1.34 °C to −2.60 ± 1.84 °C after 1 week of frozen storage, remained relatively stable for the next 3 weeks, and then rose sharply (from −1.75 ± 0.07 °C to −1.15 ± 0.64 °C). Sample with GCAFP exhibited no significant change in the first 2 weeks (from −3.25 ± 1.34 °C to −2.95 ± 0.07 °C). Except for week 3, T_m,δ_ of the control group gradually declined with extended frozen storage (from 6.85 ± 1.34 °C to 4.15 ± 3.32 °C), while T_m,δ_ of the GCAFP group remained stable before and after frozen storage (from 7.30 ± 1.98 °C to 6.56 ± 1.26 °C). At the end of frozen storage, T_m,o_ of the control group was higher than that of the GCAFP group. The upward shift of the melting peak toward higher temperatures reflects changes in ice crystal size and distribution during storage, which are associated with the deterioration of the gluten network structure. The introduction of GCAFP effectively slowed ice crystal recrystallization and the associated structural degradation, thereby preserving gluten protein quality.

Similar to previous studies, the melting curves of all samples exhibited a gradual shift. The melting curve of the control group (Figure 2A) gradually shifted toward a higher temperature region, displaying more pronounced displacement than the GCAFP group (Figure 2B), while the GCAFP group exhibited only minor fluctuations. By integrating and analyzing each melting curve, the relevant melting parameters were obtained, as summarized in Table 3. As the number of freeze–thaw cycles increased, T_m,o_ of the control group rose significantly, and the melting curve shift to the high-temperature region reflected the transformation of water from non-freezing to freezable. This indicated that gluten protein temperature in the control group exhibited greater displacement toward the high-temperature region, with substantial changes in water fluidity, leading to increased ice crystal recrystallization and the formation of large ice crystals. Conversely, T_m,o_ of the GCAFP group remained consistently lower than that of the control group under identical storage conditions, suggesting that GCAFP reduced the transition of non-freezable to freezable water.

In the control group, T_m,δ_ gradually decreased, while ΔH_m_ and F_w_ progressively increased with the number of freeze–thaw cycles. This pattern likely resulted from ice crystal formation and recrystallization, which damaged the gluten network, exposed hydrophobic groups, and caused the separation of bound water, leading to increased water fluidity. As water in the wet gluten crystallized, part of the semi-bound water migrated due to weakened polymer bindings. Small ice crystals aggregated into larger ones, piercing the gluten network, reducing water retention, and causing greater water loss, thereby increasing F_w_. In contrast, after five freeze–thaw cycles, T_m,δ_ of the GCAFP group remained significantly greater than that of the control group, while ΔH_m_ and F_w_ decreased by 1.55% and 5.19%, respectively. The presence of GCAFP slowed the decline of T_m,δ_ and the rise of ΔH_m_ and F_w_, demonstrating that GCAFP effectively reduced the rate of structural deterioration during repeated freezing and thawing.

In summary, GCAFP addition mitigated the F_w_ increase and improved the freezing stability of wet gluten protein during frozen storage and repeated freeze–thaw cycles. Bhattacharya et al. suggested that reduced water migration and lower F_w_ in frozen dough are correlated with gluten protein damage when first exposed to extremely low temperatures. Similarly, Kontogiorgos et al. proposed that frozen storage exposed hydrophobic groups in gluten protein, promoting dehydration, water release, and consequently an increase in F_w_ [8]. The hydrophilic groups in GCAFP bind to water molecules, restricting their mobility and thereby reducing F_w_ in wet gluten protein.

### 3.2. Effects of GCAFP on Water Fluidity of Wet Gluten Protein

**Figure 3:**
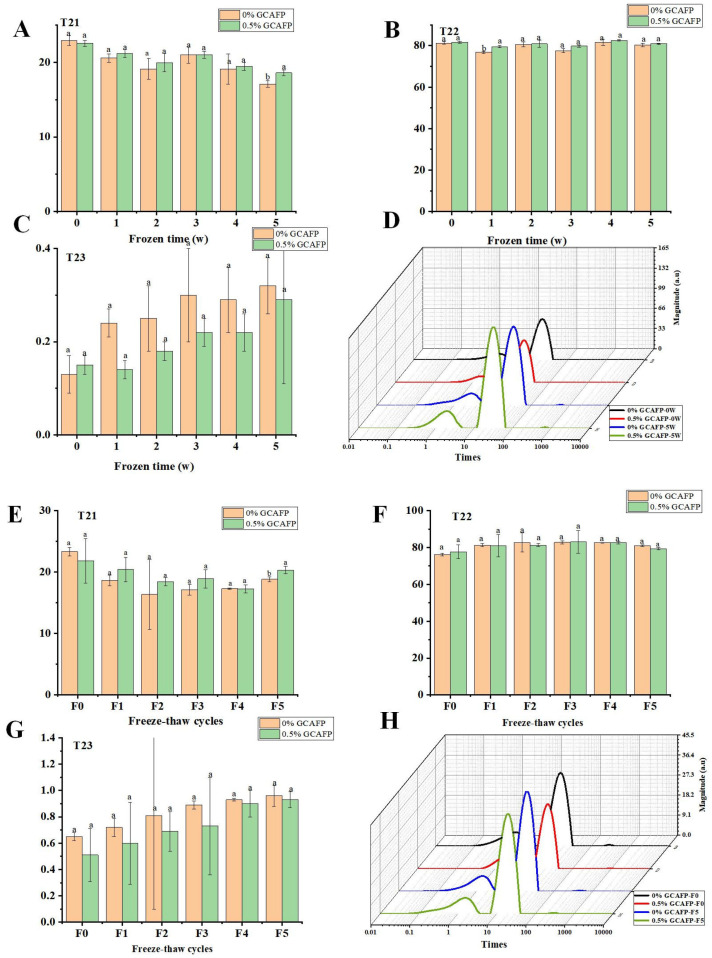
Effect of GCAFP on wet gluten T2 relaxation time (T21, T22 and T23). (**A**) Change in peak proportion of T21 after freeze 0, 1, 2, 3, 4, 5 W; (**B**) Change in peak proportion of T22 after freeze 0, 1, 2, 3, 4, 5 W; (**C**) Change in peak proportion of T23 after freeze 0, 1, 2, 3, 4, 5 W; (**D**) Typical distribution of T2 relaxation of wet gluten; (**E**) Change in peak proportion of T21 after freeze–thaw treatment 0, 1, 2, 3, 4, 5 cycles; (**F**) Change in peak proportion of T22 after freeze–thaw treatment 0, 1, 2, 3, 4, 5 cycles; (**G**) Change in peak proportion of T23 after freeze–thaw treatment 0, 1, 2, 3, 4, 5 cycles; (**H**) Typical distribution of T2 relaxation of wet gluten sample. Note: GCAFP: Grass carp antifreeze peptide.

Understanding the behavior of water within gluten protein under different storage and processing conditions is crucial for improving the quality and stability of dough. In this context, the T2 relaxation time distribution curve was used to analyze water dynamics within gluten protein subjected to freezing and freeze–thaw cycles. According to Figure 3, the typical T2 relaxation time distribution of wet gluten protein was divided into three proton peaks: T21 (0.03–10 ms), T22 (10–90 ms), and T23 (110–900 ms). Based on the binding strength of water molecules to food macromolecules, T21, T22, and T23 correspond to bound water, semi-free water, and free water, respectively [26,27]. After frozen storage, T21 in the control sample decreased, T22 increased initially and then declined slightly, and T23 increased. After 5 weeks of frozen storage, the reduction in T21 and rise in T23 indicated that frozen storage treatment increased water fluidity in wet gluten protein. This might result from ice crystal recrystallization, which disrupted the gluten structure and reduced protein cross-linking, thereby weakening the water-binding ability of gluten [28]. In contrast, GCAFP prevented the decrease of T21 and increase of T23 in the wet glutein protein, indicating that GCAFP addition mitigated the effects of freezing and frozen storage on water fluidity, consistent with previous studies [17,29].

Following freeze–thaw cycles, T21 in the control group decreased sharply before slightly increasing, T22 increased slightly, and T23 declined slightly. The decrease in T21 and the increase in T23 indicated that freeze–thaw cycles shifted water in wet gluten protein toward greater mobility. This resulted from ice crystal recrystallization induced by repeated freeze–thaw cycles, which disrupted gluten cross-linking and weakened its ability to bind water [28]. After 5 freeze–thaw cycles, T21 in the GCAFP group remained higher, while T23 was lower than in the control, demonstrating that GCAFP reduced water fluidity and alleviated the adverse effects of freeze–thaw cycles on the gluten protein network.

### 3.3. Effects of GCAFP on Water Distribution of Wet Gluten Protein

**Figure 4:**
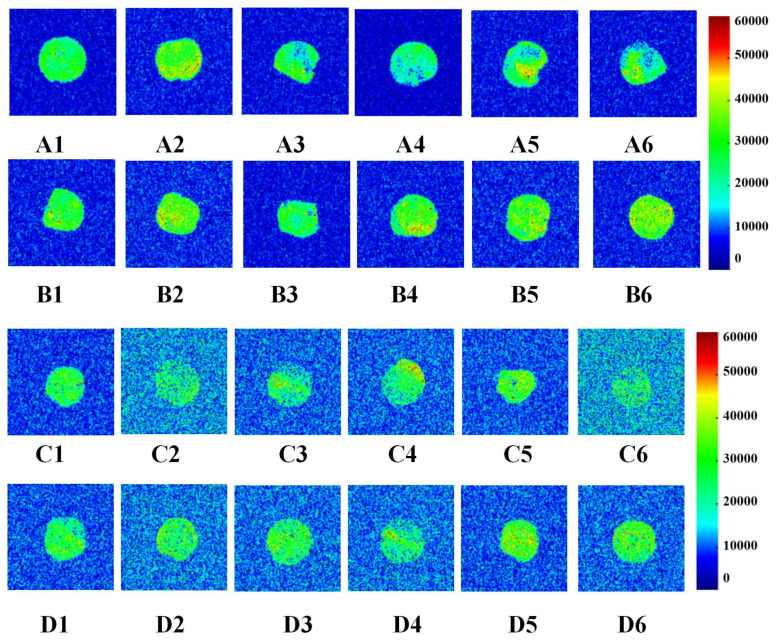
Change in water distribution of wet gluten (the colors blue through bright yellow to red represent increasing moisture contents in wet gluten samples). A1, A2, A3, A4, A5 and A6 were the water distribution after 0, 1, 2, 3, 4, 5 W frozen storage of 0% GCAFP gluten protein; B1, B2, B3, B4, B5 and B6 were the water distribution after 0,1,2,3,4,5 W frozen storage of 0% GCAFP gluten protein; C1, C2, C3, C4, C5 and C6 were the water distribution after 0, 1, 2, 3, 4, 5 freeze–thaw cycles of 0% GCAFP gluten protein; D1, D2, D3, D4, D5 and D6 were the water distribution after 0, 1, 2, 3, 4, 5 freeze–thaw cycles of 5% GCAFP gluten protein. Note: GCAFP: Grass carp antifreeze peptide.

Low-field nuclear magnetic resonance (LF-NMR) imaging was used to visualize changes in water distribution in wet gluten protein during frozen storage and freeze–thaw cycles. This non-destructive technique measures proton density, where brighter regions indicate higher water content and darker regions indicate lower water content (Figure 4).

#### 3.3.1. Water Distribution During Frozen Storage

As frozen storage time increased (0–5 weeks), notable differences were observed between the control and GCAFP samples. In the control, uneven proton signals appeared after 2 weeks, and edge clarity gradually diminished at 4 and 5 weeks, indicating water migration and structural deterioration. By contrast, GCAFP samples maintained clear boundaries and uniform proton signal intensity throughout storage, indicating that GCAFP addition promoted retain water more evenly within the gluten matrix. This result aligned with the findings by Ding et al. [29] who reported that antifreeze proteins from barley improved water distribution in frozen gluten systems.

#### 3.3.2. Water Distribution During Freeze–Thaw Cycles

After repeated freeze–thaw cycles (F0–F5), the control sample exhibited a pronounced decrease in proton signal intensity, implying serious water loss and damage to the gluten network. In contrast, GCAFP exhibited a much slower decline, indicating enhanced water-holding capacity. This can be attributed to the strong hydrophilicity of GCAFP and its ability to form extensive hydrogen bonds with gluten molecules, reinforcing the protein network and improving viscosity. Moreover, GCAFP limited ice crystal recrystallization, thereby reducing the amount of free water formed during thawing at 32 °C. These results further confirm that GCAFP effectively mitigates the deterioration of gluten structure and moisture distribution caused by repeated freezing and thawing [29].

### 3.4. Effects of GCAFP on Rheological Properties of Wet Gluten Protein

**Figure 5:**
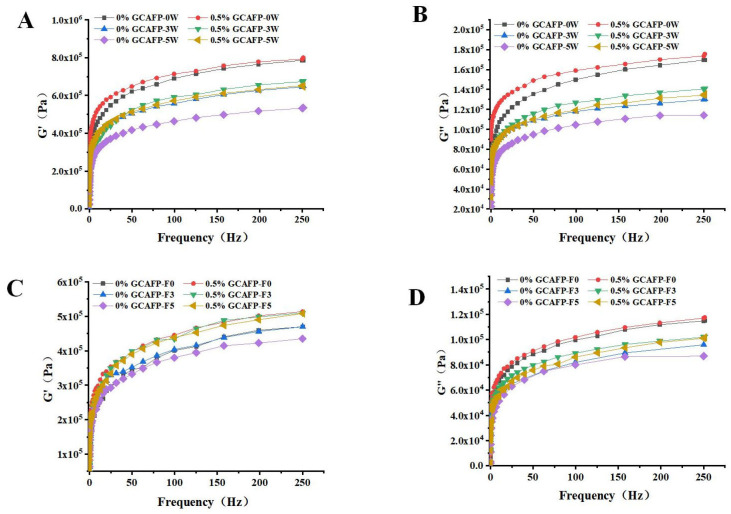
Effect of GCAFP on G’ and G” of wet gluten protein during frozen storage and freeze–thaw cycles. (**A**) The G’ of wet gluten after freeze treatment; (**B**) The G” of wet gluten after freeze treatment; (**C**) The G’ of wet gluten after freeze–thaw cycles; (**D**) The G” of wet gluten after freeze–thaw cycles. Note: GCAFP: Grass carp antifreeze peptide.

It was believed that weakening of the cross-linking of gluten proteins caused by the mechanical stress of ice crystal growth and recrystallization on the gluten network would inevitably damage the rheological properties of gluten proteins during freezing Therefore, the effects of GCAFP on G’ and G” in frozen wet gluten protein during frozen storage and freeze–thaw cycles were analyzed using a dynamic rheometer. Similar to frozen dough, G’ and G” of wet gluten protein decreased with increasing frozen storage time and freeze–thaw cycles. As shown in Figure 5, GCAFP had no significant impact on G’ and G” in fresh wet gluten protein. However, GCAFP could G’ and G” values in the sample with remained higher than those in the control group at the same frozen storage cycle and number of freeze–thaw cycles. After 5 weeks of frozen storage, G’ and G” curves in the GCAFP group were significantly higher than those in the control group after 3 weeks of frozen storage. Jiang et al. reported that changes in dynamic rheological properties of gluten protein during frozen storage were primarily attributed to ice crystal growth and recrystallization, which exerted mechanical pressure on the gluten network and weakened gluten protein cross-linking [30]. GCAFP addition effectively inhibited ice crystal growth and recrystallization, reducing mechanical pressure on the gluten network and protecting wet gluten protein. Wang et al. found that changes in rheological properties of gluten protein during frozen storage were related to molecular weight and protein structure. Yanjie Zhang et al. found that the addition of AsAFP can decrease the ice damage to hydrated gluten and slow down the mechanical damage to the gluten matrix caused by ice crystal growth and recrystallization [5].

As shown in Figure 5, when wet gluten protein was scanned across a frequency range of 0–250 Hz, G’ and G” gradually increased, but the elastic modulus remained greater than the viscous modulus. Thus, the wet gluten protein system retained its viscoelastic nature after frozen storage, aligning with previous studies [31,32]. Both G’ and G” in the wet gluten protein system gradually decreased during frozen storage, with G’ declining more sharply. This decline may have resulted from water molecule redistribution, secondary protein structure alterations, and protein degradation. Past studies indicate that glutenin in wheat gluten primarily contributes to elasticity, while gliadin mainly imparts viscosity [31,33]. G’ varied significantly with increasing frozen storage time, suggesting that ice crystal formation during frozen storage caused greater wheat gluten breakage. At the same frozen storage duration, GCAFP addition relatively increased G’ and G” in the wet gluten protein system, demonstrating that GCAFP may mitigate glutenin and gliadin degradation by inhibiting water redistribution, secondary structure changes, and protein degradation. It is noteworthy that although GCAFP addition resulted in higher G’ and G” values, this does not necessarily imply a stiffer or overly rigid gluten structure. The elevated moduli primarily reflect the improved integrity and continuity of the gluten network, which counteracts the weakening effects of ice crystal growth and protein denaturation. Within the first 5 weeks of frozen storage, the viscoelastic balance (G’/G” ratio) remained within the typical range reported for fresh or mildly frozen gluten systems, indicating that GCAFP preserved the natural elasticity without causing excessive hardness. This finding is consistent with the protective mechanism of antifreeze peptides, which stabilize protein–water interactions and maintain structural flexibility under freezing conditions.

Table 4 presented G’ and G” variations in the wet gluten protein system as a function of frozen storage time and GCAFP addition at a scanning frequency of 99.581 Hz. Wet gluten protein samples subjected to frozen storage exhibited significantly lower G’ and G” compared to fresh samples (0 weeks). Kline and Ribotta also reported that freezing reduced G’ and G” [28,34]. Jia similarly observed that freezing decreased G’ and G” in the gluten protein system [24]. However, the addition of Ligustrum lucidum Ait. antifreeze protein TSISP mitigated G’ and G” reductions, protecting glutenin and gliadin from frozen storage-induced degradation. These changes in wet gluten protein rheology during freezing and frozen storage may be closely linked to secondary structure modifications and glutenin subunit degradation. Under identical frozen storage conditions, G’ and G” values in the 0.5% GCAFP group remained significantly higher than in the control group at a scanning frequency of 99.581 Hz, suggesting that GCAFP influenced gluten protein secondary structure and molecular weight distribution.

### 3.5. Effects of GCAFP on the Structure of Wet Gluten Protein

The formation and stability of the gluten protein network are closely linked to its structural characteristics. Therefore, elucidating how GCAFP influences the structural organization of gluten is essential for understanding its role in maintaining gluten integrity under stress conditions like frozen storage and freeze–thaw cycles. To gain deeper insight into these effects, the influence of GCAFP on the free thiol group content, secondary structure, electrophoretic bands, and the distribution of relative molecular weight in wet gluten protein was systematically investigated.

#### 3.5.1. Effects of GCAFP on the Distribution of Relative Molecular Weight of Gluten Protein

**Figure 6:**
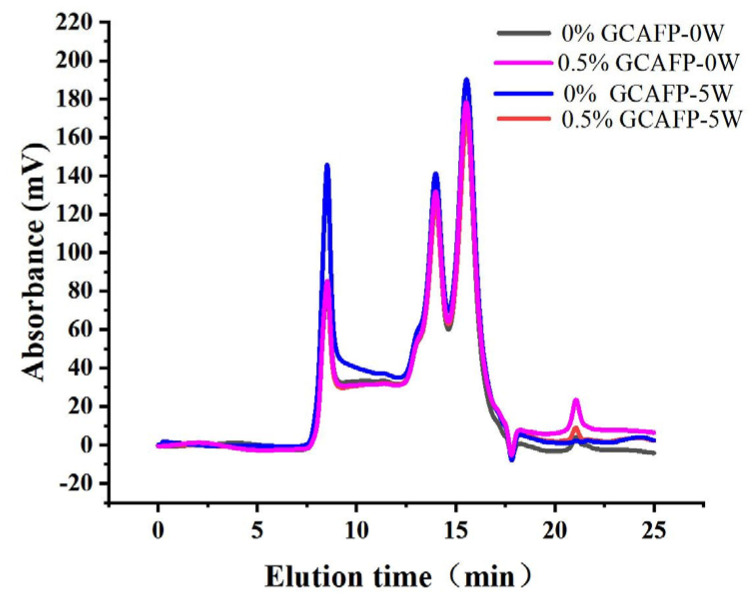
Effect of GCAFP on the molecular weight distribution of wet gluten. Note: GCAFP: Grass carp antifreeze peptide.

The relative molecular weight of wheat gluten protein was analyzed using the SE-HPLC method [35]. Previous studies have shown that protein polymerization degree can be indirectly assessed by its solubility in SDS [36]. Figure 6 presents the SE-HPLC spectra of wet gluten protein following frozen storage. According to relative molecular weight (M_w_), the chromatograms were divided into four fractions: high molecular weight gluten aggregates (F1, M_w_ 370,000–688,000), medium molecular weight gluten aggregates (F2, Mw 91,000–370,000), monomeric and alcohol-soluble proteins (F3, M_w_ 16,000–91,000), and peptide chains and amino acids (F4, M_w_ < 10,000) [37]. F1 and F2 fractions correspond to the SDS-soluble polymer protein region (SDS-P), mainly composed of glutenin with minor amounts of gliadin, while F3 and F4 fractions represent the monomer protein region (SDS-M), consisting primarily of gliadin.

Although no obvious change in the molecular weight distribution of SDS-soluble gluten protein was observed during frozen storage (Figure 6), the relative content of gluten proteins with different molecular weights and the degree of glutenin macropolymer (GMP) depolymerization changed significantly (Table 5). After freezing treatment and 5 weeks of frozen storage, SDS-P and SDS-M contents in the 0.5% GCAFP group decreased compared with the control, resulting in an increased GMP content. Following 5 weeks of frozen storage, the levels of GMP depolymerization were 24.85% in the control group and 18.95% in the 0.5% GCAFP group, respectively. Sharadanant et al. reported that ice crystal recrystallization and water redistribution during freezing and frozen storage were key factors contributing to increased SDS-soluble protein content and GMP depolymerization [38]. Singh et al. stated that GMP chain length and relative molecular weight determine the number of entanglement points among GMP molecules, which are closely related to gluten protein resistance to deformation [39]. Ice crystal formation and recrystallization during frozen storage can disrupt these entanglement points, thereby weakening gluten viscoelasticity. Wheat bran AFP helped mitigate the depolymerizing of GMP, which allowed the gluten network structures in the frozen dough to become more uniform and less deformed [40]. In summary, GCAFP effectively protected gluten protein during frozen storage by reducing ice crystal–induced mechanical damage and slowing the depolymerization of GMP. This preservation of macromolecular integrity contributes to improved gluten network stability and frozen dough quality.

#### 3.5.2. Effects of GCAFP on Wet Gluten Protein Electrophoretic Band

**Figure 7:**
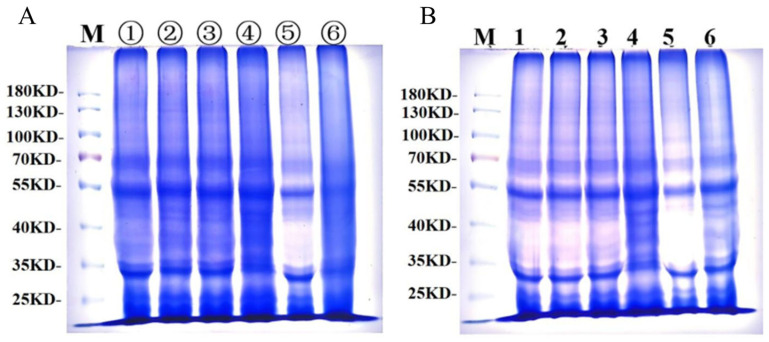
SDS-PAGE of wet gluten protein during frozen storage and freeze–thaw cycles; (**A**) SDS-PAGE of wet gluten protein during frozen storage, lanes ①–③ contained electrophoresis bands of 0.5% GCAFP frozen gluten for 0, 3, 5 W, and lanes ④–⑥ contained electrophoresis bands of 0% GCAFP frozen gluten for 0, 3, 5 W; (**B**) SDS-PAGE of wet gluten protein during freeze–thaw cycles, lane 1–3 contained electrophoresis bands of 0.5% GCAFP frozen gluten after freezing and thawing 0, 3 and 5 times, and lane 4–6 contained electrophoresis bands of 0% GCAFP frozen gluten after freezing and thawing 0, 3 and 5 times.

Figure 7A presents gluten protein changes during frozen storage, and Figure 7B shows gluten protein changes during freeze–thaw cycles. Under both frozen storage and freeze–thaw cycles, three distinct electrophoretic bands were observed between 130 and 100 kDa, indicating that protein subunits in this range have the largest molecular weight, the slowest migration rate, and lighter band intensity, corresponding to HMW-GS. Between 30 and 70 kDa, electrophoretic bands appeared darker, indicating higher protein content, faster migration rates, and lower molecular weight, corresponding to LMW-GS and alcohol-soluble proteins. Both electrophoretic images revealed no significant changes in the number of electrophoretic bands or relative mobility, demonstrating that frozen storage and freeze–thaw cycles did not significantly affect frozen dough protein subunits. Similarly, GCAFP addition had no significant impact on frozen dough protein subunits.

#### 3.5.3. Effects of GCAFP on the Content of Free Thiol Group in Wet Gluten Protein

During the formation of the gluten protein network structure, HMW-GS and LMW-GS form GMP through S-S bonds [20]. Thus, S-S cross-linking serves as the primary mechanism governing gluten network formation, and S-S bond content is a key indicator of gluten protein network formation, and its content serves as a key indicator of protein aggregation and structural stability. Changes in free thiol group content effectively reflect variations in S–S bonds within the gluten protein. In this study, the contents of S-H and S-S content in gluten protein were determined before and after frozen storage and freeze–thaw cycles.

**Figure 8:**
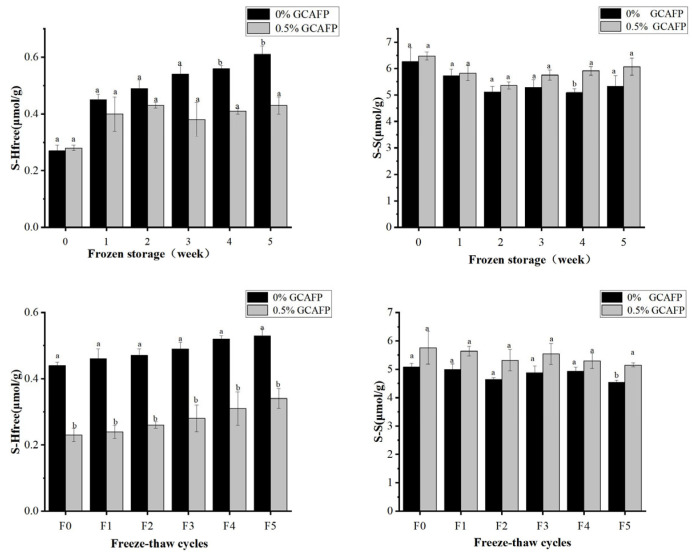
Effect of GCAFP on free thiol group content of wet gluten protein during frozen storage and freeze–thaw cycles. (**A**) Change in the free thiol group during frozen storage; (**B**) Change on the free thiol group during freeze–thaw cycles; (**C**) Change in S–S during frozen storage; (**D**) Change on the S-S during freeze–thaw cycles. Note: Means with the same letter for the same protein in the figure showed no significant difference (*p* ≥ 0.05). The results are presented as mean ± standard deviation. Note: GCAFP: Grass carp antifreeze peptide.

Figure 8A,B illustrates changes in free thiol group content in wet gluten protein during frozen storage and freeze–thaw cycles. GCAFP addition had no significant effect on free thiol content prior to frozen storage. However, during frozen storage and freeze–thaw cycles, free thiol group content increased markedly, particularly during frozen storage, indicating the breakdown of disulfide bonds in wet gluten protein. This was likely caused by water redistribution and the mechanical stress induced by ice crystal formation and recrystallization. Notably, the GCAFP-treated samples exhibited significantly lower free thiol content than the control group.

As shown in Figure 8A,C, prolonged frozen storage led to a gradual increase in free thiol content and a corresponding decrease in disulfide bond content, suggesting progressive disulfide bond degradation, which is consistent with previous studies [41]. After 5 weeks of frozen storage, the free thiol content increased by 0.34 μmol/g in the control group, whereas the increase was only 0.19 μmol/g in the 0.5% GCAFP group. Throughout the entire storage period (0–5 weeks), the free thiol content in the control group steadily increased, whereas free thiol content in wet gluten protein of the 0.5% GCAFP group remained significantly lower than in the control group. Similarly, during frozen storage (0–5 weeks), disulfide bond content in the control group declined. However, at the same frozen storage time, disulfide bond content in the 0.5% GCAFP group remained higher than in the control group. Comparative analysis indicated that thiol and disulfide bond changes in wet gluten protein of the 0.5% GCAFP group were smaller than those in the control group, demonstrating that GCAFP effectively reduced disulfide bond degradation during frozen storage, thereby preserving the gluten protein network structure. Similarly, Figure 8B,D illustrates that with an increasing number of freeze–thaw cycles, free thiol group content increased while disulfide bond content decreased. After 5 freeze–thaw cycles, the free thiol content increased by 0.19 and 0.11 μmol/g in the control and 0.5% GCAFP groups, respectively. Correspondingly, the disulfide bond content declined less in the GCAFP group than in the control, demonstrating that GCAFP addition slowed S–S bond degradation during repeated freezing and thawing.

Taken together, the results indicate that GCAFP positively stabilized the disulfide bonds and suppressed the formation of free thiols in wet gluten protein during frozen storage and freeze–thaw cycles. This protective effect helps preserve the gluten network structural integrity and contributes to improved dough quality under freezing conditions.

#### 3.5.4. Effects of GCAFP on the Secondary Structure of Wet Gluten Protein

The relative proportions of different secondary protein structures were obtained by peak-fitting infrared spectrum data and calculating the peak area (Table 6). The secondary structure of wet gluten protein primarily consists of β-sheet structures (50.56%), followed by α-helix (19.32%), β-turn (20.21%), and random coil (9.91%). After frozen storage, significant changes occurred in the secondary structure of gluten protein. The β-sheet content increased, whereas the α-helix and random coil contents declined. After 5 weeks of frozen storage, the α-helix content decreased by 3.61%, β-sheet content increased by 8.92%, β-turn content decreased by 1.83%, and random coil content declined by 3.48% in the wet gluten protein. These results indicated that α-helix was particularly sensitive to low-temperature conditions, with some α-helix structures transforming into β-sheet and β-turn configurations during freezing, consistent with previous studies [10,23]. After the addition of 0.5% GCAFP to the wet gluten protein, α-helix content decreased by only 2.89%, β-sheet content increased by only 7.37%, β-turn content decreased by only 2.41%, and random coil content declined by only 2.07% after 5 weeks of frozen storage. These data indicated that GCAFP addition mitigates α-helix to β-turn conversion caused by low temperatures during frozen storage, preserving gluten protein secondary structure from ice crystal damage, consistent with previous studies [22].

The relative proportions of different secondary protein structures during freeze–thaw cycles, obtained from peak area calculations of infrared spectrum fitting for frozen wet gluten protein with GCAFP, were summarized in Table 7. In wet gluten protein without freeze–thaw treatment, the secondary structure is dominated by β-sheet (49.76%), followed by α-helix (20.08%), β-turn (19.36%) and random coil (10.80%). After freeze–thaw cycles, significant changes occurred in the gluten protein secondary structure. In the control group, the β-sheet content increased, whereas α-helix and random coil decreased, while β-turn remained relatively stable (consistent with frozen storage trends). After 5 cycles, the control showed α-helix decreased by 5.41%, β-sheet increased by 8.58%, β-turn decreased by 1.33%, and random coil decreased by 1.74%. In the 0.5% GCAFP group, α-helix content decreased by only 4.87%, β-sheet content increased by only 6.76%, β-turn content increased by only 1.82%, and random coil structure content declined by only 0.07%. These results indicated that GCAFP mitigates structural transitions caused by freeze–thaw recrystallization, thereby reducing hydrogen bond disruption in gluten secondary structures [10].

Jood et al. reported that during freezing, ice crystal formation driven by water molecule migration disrupts hydrogen bonds in the secondary protein structure, exposing hydrophilic and hydrophobic regions and altering intra- and intermolecular cross-linking, which ultimately modifies the secondary conformation of proteins [42]. Compared with the control group, the decrease in α-helix content and the increase in β-sheet content in the wet gluten protein system with GCAFP addition after frozen storage and freeze–thaw cycles were less pronounced. These results suggest that GCAFP inhibits ice crystal formation and recrystallization, thereby mitigating hydrogen bond disruption and preventing protein molecular structure alterations. Additionally, Liu et al. reported that gluten protein consists of two components: a poorly soluble fraction and a soluble fraction, which exhibit distinct structural changes during frozen storage. In the poorly soluble fraction, β-sheet structures transition to β-turn, α-helix, and random coil, whereas in the soluble fraction, α-helix structures convert into β-sheet and β-turn [43]. Based on the observed variations in secondary structure ratio changes in gluten protein following frozen storage and freeze–thaw cycles, structural modifications in both insoluble and soluble gluten fractions contribute to the observed secondary structure variations. GCAFP addition enhanced intramolecular hydrogen bonding and promoted the reformation of disrupted intermolecular hydrogen bonds, thereby stabilizing the secondary structure of gluten protein during repeated freezing and thawing.

#### 3.5.5. Effects of GCAFP on Protein Microstructure of Wet Gluten

**Figure 9:**
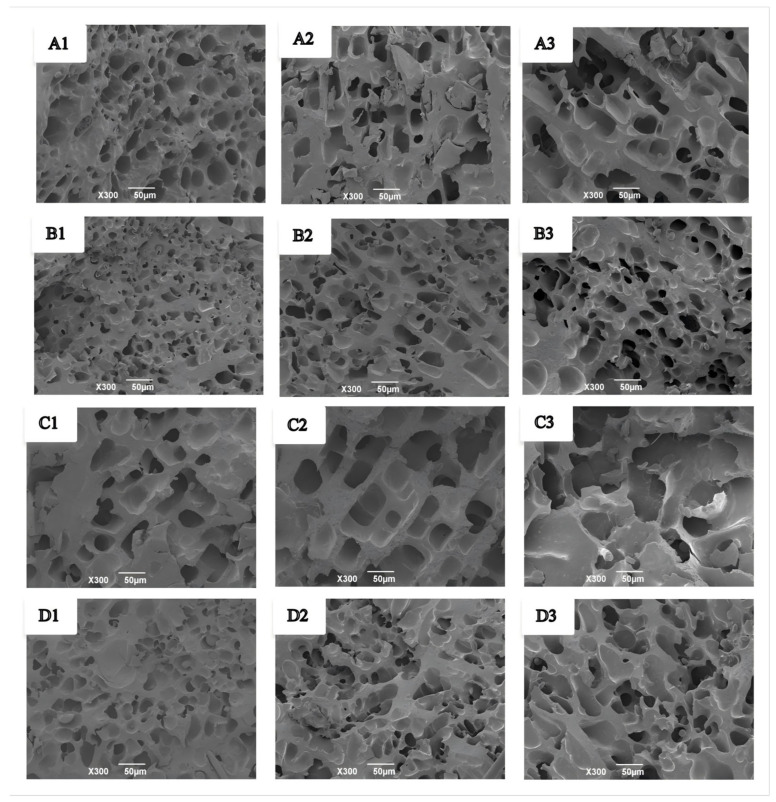
Effect of GCAFP on the microstructure of wet gluten protein during frozen storage and freeze–thaw cycles. (**A1**–**A3**) were 0% GCAFP wet gluten protein after 0, 3, and 5 W of frozen storage; (**B1**–**B3**) were 5% GCAFP wet gluten protein after 0, 3, and 5 W of frozen storage; (**C1**–**C3**) were 0% GCAFP wet gluten protein after 0, 3, and 5 freeze–thaw cycles; (**D1**–**D3**) were 5% GCAFP wet gluten protein after 0, 3, and 5 freeze–thaw cycles. Note: GCAFP: Grass carp antifreeze peptide.

The microstructural changes of wet gluten protein during frozen storage and freeze–thaw cycles are shown in Figure 9. Fresh wet gluten protein exhibited smaller, evenly distributed pores and a compact network structure (Figure 9(A1,B1)), and the addition of GCAFP further enhanced network compactness. During frozen storage, the gluten network underwent varying degrees of structural damage. After 3 and 5 weeks, pores in both the control and GCAFP groups enlarged and became unevenly distributed (Figure 9(A2,A3)). However, in the GCAFP group, pores remained more uniform and smaller in size compared to the control (Figure 9(B2,B3)). These observations were consistent with microstructural changes observed in frozen dough. Ice recrystallisation and cryoshrinkage during temperature fluctuations can decrease the interstitial regions between adjacent ice crystals, leading to mechanical damage to the gluten network [44]. Overall, GCAFP addition mitigated structural damage to wet gluten protein during frozen storage, consistent with previous studies [45].

Similarly, during freeze–thaw cycles, fresh wet gluten protein exhibited smaller, uniform pores and a compact structure (Figure 9(C1,D1)), which were further compacted by GCAFP. With repeated freeze–thaw cycles, pores enlarged and became more irregular in both control and GCAFP groups (Figure 9(C2,C3)). However, the GCAFP group remained smaller and more uniformly distributed pores after 3 and 5 freeze–thaw cycles (Figure 9(D2,D3)), demonstrating that GCAFP effectively preserved the microstructural integrity of gluten protein during freeze–thaw stress.

These structural changes can be attributed to ice crystal dynamics. During frozen storage and freeze–thaw cycles, small ice crystals gradually grow and merge into larger crystals due to temperature fluctuations, while sublimation of ice crystals forms large voids. Expansion of these large ice crystals exerts pressure on the gluten protein network, which can weaken or even disrupt disulfide bonds and compromise network integrity. GCAFP addition inhibited ice crystal growth and recrystallization, reduced the formation of large ice crystals, and maintained a more uniform and compact gluten network with smaller pores, thereby enhancing gluten structural stability under freezing conditions.

## 4. Conclusions

This study investigated the effects of antifreeze peptides from grass carp (GCAFP) on the thermodynamic characteristics, water state, rheological properties, structure, and microscopic morphology of wet gluten protein. The findings demonstrated that GCAFP significantly slowed gluten system degradation and preserved gluten network integrity during frozen storage and freeze–thaw cycles. Based on the thermodynamic properties of the gluten protein, GCAFP effectively mitigated changes in apparent specific heat and F_w_. GCAFP improved the freeze–thaw characteristics of fresh wet gluten protein by lowering the freezing temperature and expanding the melting zone. Additionally, free thiol group measurements revealed that GCAFP significantly inhibited free thiol group content increase in wet gluten protein, thereby enhancing disulfide bond stability at low temperatures. Molecular weight distribution analysis indicated that GCAFP reduced GMP depolymerization by inhibiting gluten protein recrystallization during frozen storage and freeze–thaw cycles. This optimization of the rheological properties of wet gluten protein and pre-fermented frozen dough ultimately improved the quality of pre-fermented frozen Mantou.

Overall, GCAFP positively influenced the thermodynamic characteristics, water status, rheological properties, structure, and microscopic morphology of the wet gluten protein system. Its antifreeze effect was comparable to that of winter wheat antifreeze protein, privet leaf antifreeze protein, oat antifreeze protein, pig skin gelatin, salmon antifreeze peptide, and other antifreeze proteins and peptides. However, compared with these antifreeze proteins, GCAFP offers broader availability, lower cost, higher safety, and better application potential. Thus, GCAFP holds great promise for use in food and related industries.

However, it should be noted that GCAFP, being derived from grass carp, is not suitable for vegetarian or vegan food products. In addition, although GCAFP showed no adverse effects on the physicochemical characteristics of gluten protein in this study, its influence on the sensory properties of finished products such as bread and steamed Mantou still requires further investigation. Future studies should therefore focus on evaluating its sensory impact, allergenicity, and compatibility with plant-based or alternative protein systems to expand its application range.

## Figures and Tables

**Table 1:** Effects of GCAFP on freeze–thaw parameters and F_w_ of wet gluten protein.

Gluten Protein	0% GCAFP	0.5% GCAFP
T_f_ (°C)	−8.50 ± 1.31 a	−10.75 ± 2.49 a
T_m,o_ (°C)	−1.70 ± 0.41 a	−2.05 ± 0.37 a
T_m,p_ (°C)	−0.65 ± 0.28 a	−1.15 ± 0.04 a
T_m,δ_ (°C)	3.60 ± 1.4 a	5.65 ± 0.12 b
∆H_m_ (J/g)	145.7 ± 0.54 a	113.9 ± 1.38 b
F_w_ (%)	81.04 ± 2.65 a	61.28 ± 1.02 b

Note: Means with the same letter in the same line showed no significant difference (*p* ≥ 0.05). The results are presented as mean ± standard deviation. T_f_: the peak temperature of the freezing curve; T_m,o_: the onset temperature of the heat absorption curve; T_m_,_p_: the peak temperature of the melting curve; T_m,δ_: Melting temperature range; ∆H_m_: Melting enthalpy; F_w_: the ratio of frozen water to total water.

**Table 2:** Effects of GCAFP on melting parameters of frozen hydrated gluten after freeze treatment.

Gluten Protein	Frozen Storage Time (W)	T_m,o_ (°C)	T_m,δ_ (°C)	∆H_m_ (J/g)	W_A_ (%)	F_w_ (%)
0% GCAFP	0	−3.35 ± 1.34 a	6.85 ± 1.34 b	96.91 ± 2.47 b	53.90 ± 0.92 a	57.75 ± 0.09 b
	1	−2.60 ± 1.84 a	4.80 ± 4.36 a	102.07 ± 3.64 a	51.94 ± 0.42 a	58.83 ± 1.43 b
	2	−1.75 ± 0.07 a	6.83 ± 0.72 b	123.95 ± 13.20 a	51.37 ± 11.38 a	69.52 ± 17.56 ab
	3	−1.23 ± 0.46 a	4.70 ± 3.39 a	117.14 ± 0.37 a	47.89 ± 0.84 a	73.30 ± 1.06 ab
	5	−1.15 ± 0.64 a	4.15 ± 3.32 a	124.14 ± 4.76 a	43.65 ± 0.84 a	81.89 ± 7.37 a
0.5% GCAFP	0	−3.25 ± 1.34 b	7.30 ± 1.98 a	96.08 ± 1.30 b	51.64 ± 0.63 a	54.91 ± 0.15 b
	1	−2.60 ± 1.59 b	6.23 ± 1.25 a	105.35 ± 5.21 a	48.36 ± 0.23 a	56.97 ± 17.52 b
	2	−2.95 ± 0.07 b	7.75 ± 0.21 a	108.7 ± 2.69 a	50.26 ± 1.23 a	63.96 ± 0.48 b
	3	−1.70 ± 0.20 a	7.20 ± 0.14 a	118.77 ± 7.18 a	48.66 ± 0.88 a	75.12 ± 5.26 a
	5	−1.55 ± 0.07 a	6.56 ± 1.26 a	121.80 ± 2.40 a	48.89 ± 1.07 a	78.41 ± 3.98 a

Note: Means with the same letter in the same column showed no significant difference (*p* ≥ 0.05). The results are presented as mean ± standard deviation. T_m,o_: the onset temperature of the heat absorption curve; T_m,δ_: Melting temperature range; ∆H_m_: Melting enthalpy; F_w_: the ratio of frozen water to total water.

**Table 3:** Effects of GCAFP on melting parameters of frozen hydrated gluten after freeze–thaw treatment.

Gluten Protein	Freeze–Thaw	T_m,o_ (°C)	T_m,δ_ (°C)	∆H_m_ (J/g)	W_A_ (%)	F_w_ (%)
0% GCAFP	0	−3.35 ± 1.34 b	7.85 ± 1.34 a	96.91 ± 2.47 a	53.90 ± 0.92 a	57.75 ± 0.09 b
	F0	−2.90 ± 0.71 ab	7.55 ± 0.07 a	106.22 ± 27.13 a	48.33 ± 3.01 ab	67.01 ± 21.16 ab
	F1	−2.45 ± 0.21 ab	7.00 ± 4.95 a	119.50 ± 27.73 a	47.93 ± 3.01 ab	75.34 ± 22.05 ab
	F2	−2.21 ± 0.28 ab	7.05 ± 0.78 a	117.80 ± 1.84 a	49.36 ± 0.71 ab	71.46 ± 3.40 ab
	F3	−2.20 ± 0.14 ab	7.15 ± 0.49 a	120.40 ± 4.38 a	51.84 ± 1.86 ab	70.25 ± 4.16 ab
	F4	−2.05 ± 1.06 ab	6.35 ± 1.91 a	123.41 ± 7.70 a	48.97 ± 0.95 a	71.25 ± 1.89 a
	F5	−1.90 ± 0.14 a	5.67 ± 1.27 a	122.85 ± 5.30 a	49.97 ± 1.37 a	73.59 ± 1.16 a
0.5% GCAFP	0	−3.25 ± 0.72 a	7.30 ± 1.98 a	96.08 ± 1.30 a	51.64 ± 0.63 ab	54.91 ± 0.15 b
	F0	−3.05 ± 0.49 a	7.70 ± 0.28 a	103.72 ± 23.6 a	54.93 ± 1.23 a	56.40 ± 11.60 ab
	F1	−2.90 ± 0.98 a	7.65 ± 2.47 a	104.09 ± 14.48 a	49.01 ± 0.50 b	58.54 ± 4.75 a
	F2	−2.57 ± 0.72 a	6.50 ± 1.65 a	117.20 ± 9.90 a	52.56 ± 0.18 a	69.83 ± 9.34 a
	F3	−2.35 ± 0.07 a	5.15 ± 3.32 a	118.74 ± 6.73 a	51.84 ± 1.86 ab	68.70 ± 6.35 a
	F4	−2.40 ± 1.70 a	7.05 ± 1.06 a	111.3 ± 10.89 a	50.84 ± 1.86 ab	64.44 ± 8.60 a
	F5	−2.50 ± 0.14 a	6.20 ± 1.15 a	116.25 ± 3.18 a	51.39 ± 0.66 a	68.40 ± 3.67 a

Note: Means with the same letter in the same column showed no significant difference (*p* ≥ 0.05). The results are presented as mean ± standard deviation. T_m,o_: the onset temperature of the heat absorption curve; T_m,δ_: Melting temperature range; ∆H_m_: Melting enthalpy; F_w_: the ratio of frozen water to total water.

**Table 4:** The effect of GCAFP on viscoelastic modules of 99.581 Hz of frozen hydrated gluten after frozen storage.

Gluten Protein	Frozen Storage Time (W)	G’	G”
0% GCAFP	0	571,973	149,840
	3	591,317	117,871
	5	464,525	101,221
0.5% GCAFP	0	689,859	159,039
	3	713,542	126,809
	5	556,870	119,135

Note: GCAFP: Grass carp antifreeze peptide.

**Table 5:** Effects of frozen storage on the molecular weight distribution of hydrated gluten and depolymerization degree of GMP.

Gluten Protein	Frozen Storage Time (W)	SDS-P	SDS-M	GMP Content	GMP Depolymerization Level
0% GCAFP	0	13.04	39.68	47.28	-
	3	15.45	35.21	39.34	18.91
	5	17.60	46.87	35.53	24.85
0.5% GCAFP	0	12.79	38.81	48.40	-
	3	14.25	41.90	43.85	9.40
	5	15.32	45.45	39.23	18.95

Note: GCAFP: Grass carp antifreeze peptide.

**Table 6:** Effects of GCAFP on the secondary structures of hydrated gluten after freeze treatment.

Gluten Protein	Frozen Storage Time (W)	Secondary Structure (%)			
		α-Helix	β-Sheet	β-Turn	Random Coil
0% GCAFP	0	19.32 ± 1.34 b	50.56 ± 3.24 b	20.21 ± 3.04 a	9.91 ± 1.14 a
	3	17.44 ± 3.22 a	55.54 ± 2.31 a	19.75 ± 3.08 a	7.27 ± 1.02 a
	5	15.71 ± 2.68 a	59.48 ± 2.56 a	18.38 ± 2.59 b	6.43 ± 0.81 a
0.5% GCAFP	0	19.58 ± 3.17 a	51.02 ± 4.30 a	20.4 ± 4.35 a	8.95 ± 0.96 b
	3	17.87 ± 2.65 a	54.56 ± 2.39 a	20.37 ± 2.36 a	7.20 ± 0.63 ab
	5	16.69 ± 1.98 a	58.39 ± 1.02 a	18.04 ± 3.37 a	6.88 ± 0.34 a

Note: Means with the same letter in the same column showed no significant difference (*p* ≥ 0.05). The results are presented as mean ± standard deviation. Note: GCAFP: Grass carp antifreeze peptide.

**Table 7:** Effects of GCAFP on the secondary structures of hydrated gluten after freeze–thaw treatment.

Gluten Protein	Freeze–Thaw	Secondary Structure (%)			
		α-Helix	β-Sheet	β-Turn	Random Coil
0% GCAFP	F0	20.08 ± 3.31 b	49.76 ± 3.71 b	19.361 ± 2.04 a	10.80 ± 0.51 a
	F3	18.51 ± 2.47 ab	54.24 ± 2.96 ab	18.47 ± 1.59 a	8.78 ± 0.25 a
	F5	14.62 ± 1.82 a	58.34 ± 3.85 a	17.98 ± 1.15 a	9.06 ± 0.48 a
0.5% GCAFP	F0	20.26 ± 4.26 a	50.33 ± 2.82 a	19.86 ± 2.06 a	9.55 ± 0.74 a
	F3	19.07 ± 2.84 a	53.80 ± 3.56 a	19.01 ± 1.52 a	8.12 ± 0.62 b
	F5	15.39 ± 1.63 a	57.09 ± 4.32 a	18.04 ± 0.68 a	9.48 ± 0.92 a

Note: Means with the same letter in the same column showed no significant difference (*p* ≥ 0.05). The results are presented as mean ± standard deviation. Note: GCAFP: Grass carp antifreeze peptide.

## Data Availability

The original contributions presented in the study are included in the article. Further inquiries can be directed to the corresponding author.

## Abbreviations

The following abbreviations are used in this manuscript:

AsAFP	Oat antifreeze protein
GCAFP	Grass carp antifreeze peptide
EDTA	Ethylene Diamine Tetraacetic Acid
F_w_	Freezable water content
C_app_	Apparent specific heat
T_m_	Melting temperature
T_m,δ_	Melting temperature range
T_f_	The peak temperature of the freezing curve
T_m,o_	The onset temperature of the heat absorption curve
T_m,p_	The peak temperature of the melting curve
T_m,e_	The end temperature of the melting curve
F_w_	The ratio of frozen water to total water
NMR	Nuclear magnetic resonance
M_w_	Molecular weight
SDS	Sodium dodecyl sulfonate
SDS-P	SDS-soluble polymer
SDS-I	SDS-insoluble protein
ISP	Ice structuring protein
SaAFP	Salmon antifreeze peptide
Tris-HCl	Tris (hydroxymethyl) aminomethane hydrochloride
LD	Freezing temperature
∆H_m_	Melting enthalpy
DSC	Differential scanning calorimetry
SEM	Scanning electron microscopy
DTT	Dithiothreitol
SDS-M	SDS-soluble monomeric protein
GMP	Glutenin macropolymer
